# The serum amyloid A3 promoter-driven luciferase reporter mice is a valuable tool to image early renal fibrosis development and shows the therapeutic effect of glucosyl-hesperidin treatment

**DOI:** 10.1038/s41598-019-50685-0

**Published:** 2019-10-01

**Authors:** Thanutchaporn Kumrungsee, Taishi Kariya, Kotaro Hashimoto, Takayuki Koyano, Nao Yazawa, Takao Hashimoto, Yohei Sanada, Makoto Matsuyama, Yusuke Sotomaru, Hiroaki Sakurai, Fons A. J. van de Loo, Noriyuki Yanaka

**Affiliations:** 1 Graduate School of Biosphere Science, Hiroshima University, Higashi-Hiroshima, Japan; 2 Division of Molecular Genetics, Shigei Medical Research Institute, 2117 Yamada, Minami-ku, Okayama, Japan; 3 Natural Science Center for Basic Research and Development, Hiroshima University, Hiroshima, Japan; 4 Department of Cancer Cell Biology, Graduate School of Medicine and Pharmaceutical Sciences, University of Toyama, Toyama, Japan; 5 Experimental Rheumatology, Department of Rheumatology, Radboud University Medical Center, Nijmegen, The Netherlands

**Keywords:** Imaging, Kidney diseases

## Abstract

Tubulointerstitial fibrosis is a progressive process affecting the kidneys, causing renal failure that can be life-threatening. Thus, renal fibrosis has become a serious concern in the ageing population; however, fibrotic development cannot be diagnosed early and assessed noninvasively in both patients and experimental animal models. Here, we found that serum amyloid A3 (Saa3) expression is a potent indicator of early renal fibrosis; we also established *in vivo* Saa3/C/EBPβ-promoter bioluminescence imaging as a sensitive and specific tool for early detection and visualization of tubulointerstitial fibrosis. Saa3 promoter activity is specifically upregulated in parallel with tumor necrosis factor α (TNF-α) and fibrotic marker collagen I in injured kidneys. C/EBPβ, upregulated in injured kidneys and expressed in tubular epithelial cells, is essential for the increased Saa3 promoter activity in response to TNF-α, suggesting that C/EBPβ plays a crucial role in renal fibrosis development. Our model successfully enabled visualization of the suppressive effects of a citrus flavonoid derivative, glucosyl-hesperidin, on inflammation and fibrosis in kidney disease, indicating that this model could be widely used in exploring therapeutic agents for fibrotic diseases.

## Introduction

About 750 million people suffer from chronic kidney disease (CKD) with global deaths predicted to rise from 1.2 million in 2016 to 3.1 million in 2040^1,2^. CKD causes progressive decline in both kidney function and structure without obvious outward symptoms, making it difficult to diagnose until end stage where it is irreversible and requires dialysis or kidney transplantation to maintain patients’ lives, which is an economic burden for developing countries^2–4^. Thus, effective disease assessment at early stages is essential in order to improve diagnosis and prognosis and to provide prompt treatment before the onset of end-stage renal disease. Renal fibrosis, particularly tubulointerstitial fibrosis, is a hallmark of end-stage kidney disease^5,6^. Molecular mechanisms of tubulointerstitial fibrosis involve tubular epithelial cells, a primary target of renal injurious stimuli, being injured by intratubular hydrodynamic forces, such as tubular stretch induced by obstruction; which activates inflammatory and fibrogenic signals^5–7^. Together with the alteration of tubular epithelial phenotype, these signals lead to activation of tubulointerstitial fibroblasts and myofibroblasts, resulting in deposition of extracellular matrix (ECM), such as collagen in interstitial areas, finally leading to tubulointerstitial fibrosis. At the transcriptional level, in response to injuries, transcriptional factors that regulate expression of genes involved in fibrosis, such as α-smooth muscle actin (αSMA), are activated. In addition to myofibroblasts, recent studies have suggested that renal tubular epithelial cells are an important initiator and mediator of tubulointerstitial fibrosis and attempted to isolate marker proteins in injured tubular epithelial cells to serve as indicators for early detection of tubulointerstitial fibrosis^8,9^.

In a few decades, *in vivo* imaging technologies have become essential for the basic sciences and for translational drug development because they provide an important opportunity for studying biological processes in living organisms in real time at a molecular level. Recently, imaging devices have been developed to gain high-resolution bioluminescence images of luciferase or fluorescence proteins and are applicable for studying not only tumorigenesis but also inflammatory diseases and other pathologies^10,11^. In our previous study, we successfully used a mouse serum amyloid A3 (Saa3) gene promoter-luciferase (luc) reporter to monitor inflammation in fat tissue of obese mice^12^. Saa3 is a member of the Saa family, which comprises of acute-phase proteins highly expressed under various inflammatory conditions and whose mRNA expression profiles in chronic inflammatory diseases, such as rheumatoid arthritis, atherosclerosis, and colitis, have been studied^13–15^. In the mouse Saa3 gene promoter region, there are three CCAAT/ enhancer binding protein β (C/EBPβ)-binding sites (−152, −107, and −77)^12,13,16^. C/EBPβ is transcriptionally activated by inflammatory stimuli, including inflammatory cytokines, such as interleukin-6 (IL-6), IL-1, and tumor necrosis factor α (TNF-α), and the possibility that C/EBPβ may play an important role in inflammatory signals during disease development has been explored^12,13,17–19^. In addition, there is an upregulation of the Saa family proteins in serum and kidney tissue of both patients and experimental animals having kidney disease^20–25^. Taken together, these observations indicate that C/EBPβ and its regulated Saa family of genes may represent an important target for assessing kidney injury and the Saa3-promoter reporter might be used in live animals for visualizing the injury in experimental kidney disease models and for monitoring therapeutic effects of functional food on the pathology of diseases.

Here, we report that *in vivo* Saa3/C/EBPβ-promoter bioluminescence imaging is a novel, sensitive, and specific approach for detecting and visualizing tubulointerstitial injury and fibrosis as well as monitoring the therapeutic effect of functional food on kidney disease. In fact, our *in vivo* bioluminescence imaging model revealed a functional food, which shows preventive effect on kidney disease. Importantly, we also report here that C/EBPβ plays a crucial role in renal tubulointerstitial injury and fibrosis, possibly by driving the fibrotic marker Saa3. As C/EBPβ is likely to be an upstream regulator for tubulointerstitial fibrosis, our finding may open the door to the design of future therapeutic strategies or screening for novel therapeutic functional food by controlling the levels of expression of C/EBPβ.

## Results

### Upregulation of Saa3 promoter activity in adenine-induced kidney injury

**Figure 1:**
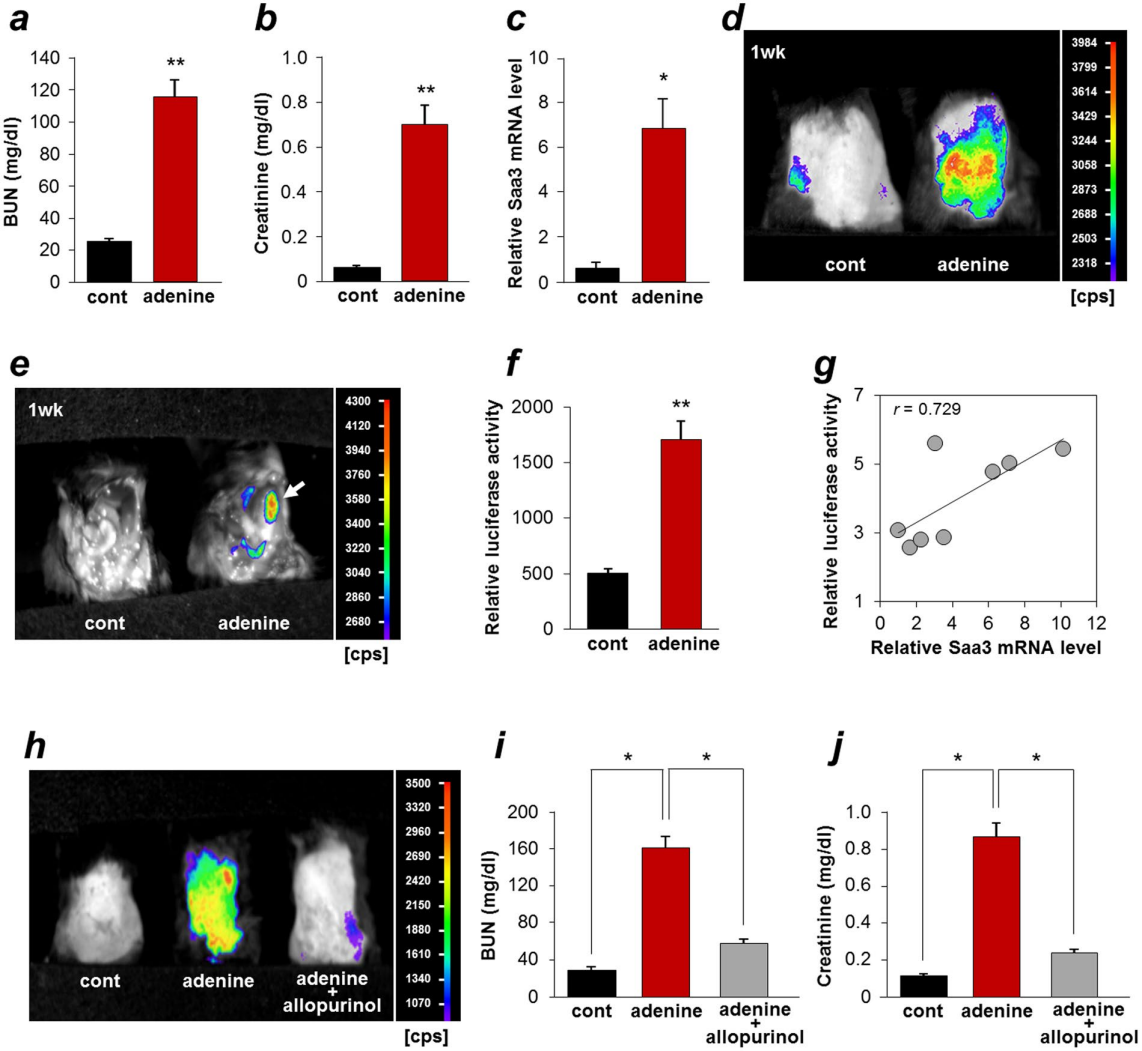
Bioluminescence imaging allows visualization of adenine-induce tubulointerstitial injury and fibrosis in Saa3 promoter-luc mice. (**a–c**) Wide-type mice were divided into two groups (*n* = 4) receiving the control diet (cont) or the adenine diet (adenine) for 3 weeks. (**a**) Plasma BUN and (**b**) creatinine levels were increased by the adenine diet (*n* = 4). (**c**) The relative Saa3 mRNA level in kidney tissues was determined by quantitative PCR and normalized to L19 mRNA level (*n* = 4). (**d**–**k**) Saa3 promoter-luc mice were divided into two groups (*n* = 4) receiving the control diet (cont) or the adenine diet (adenine) for one week. (**d**) *In vivo* bioluminescence imaging from the back of Saa3 promoter-luc mice that were fed the adenine diet shows a strong intensity of bioluminescent signal (from violet for least intense to red for most intense), reflecting kidney injury. (**e**) Exposure of mouse organs under *in vivo* bioluminescent analysis exhibited a very intense bioluminescent signal generated specifically from the injured kidney (the white arrow), and not from the adjacent organs of the mice that were fed the adenine diet. **(f**) Kidney tissues were isolated and subjected to the *ex vivo* luciferase activity assay to show enhancement of bioluminescent signal intensity induced by the adenine diet (*n* = 4). (**g**) The relative Saa3 mRNA expression level in kidney tissue was determined by quantitative PCR and normalized to L19 mRNA level (*n* = 4). Pearson’s correlation coefficient showed a positive correlation between luciferase activity (promoter activity) and Saa3 mRNA expression level in kidney tissue. (**h**–**j**) The treatment with allopurinol of Saa3 promoter-luc mice (*n* = 3) receiving the adenine diet for six weeks. (**h**) The *in vivo* bioluminescence imaging shows decreased signal intensity in the adenine-induced mouse treated with allopurinol, reflecting less severe injury in kidney, as compared with the adenine-induced mouse without allopurinol treatment. (**i,j**) Allopurinol decreased plasma BUN and creatinine levels in the adenine-induced mice (*n* = 3). All values are expressed as mean ± S.E. Student’s *t*-test, *p < 0.05, **p < 0.01.

To determine if the Saa3-promotor (−314/+50) reporter is useful for assessing kidney injury, we employed transgenic mice carrying the Saa3 promoter-luc chimeric gene. Since the transgenic mice showed high Saa3 promoter activity in kidney tissue under normal conditions (Supplementary Fig. S1a), it is logical to examine the luciferase activity based on Saa3 promoter activity during kidney disease development by *in vivo* imaging technique. In order to induce injury in kidney, mice were fed the adenine-containing diet for three weeks. We found that blood urea nitrogen (BUN) and plasma creatinine concentrations in the adenine group were significantly increased as compared to the control group (Fig. 1a,b). Pathological changes in the adenine-induced kidney were observed using hematoxylin-eosin (H&E) and Azan-Mallory (AZM) staining; deposition of 2,8-dihydroxyadenine (DHA) crystals in renal tubules (brown), expansion of interstitial ECM, and accumulation of collagen, which are typical pathologies of tubulointerstitial fibrosis, were observed after three weeks, while abnormal tubular dilation and atrophy appeared after one week of the adenine diet (Supplementary Fig. S2). We further observed that Saa3 mRNA expression was greatly upregulated (13-fold, *p* < 0.05) in the kidney tissue of adenine-fed mice (Fig. 1c). Next, to employ the *in vivo* Saa3 promoter-luc model for monitoring the kidney injury, Saa3 promoter-luc mice were divided into two groups receiving the adenine diet or the control diet and subjected to *in vivo* bioluminescent analysis. Shortly after one week of feeding, *in vivo* bioluminescence imaging from the back of Saa3 promoter-luc mice showed that injured kidneys emitted a strong bioluminescent signal (from violet for least intense to red for most intense), while uninjured kidneys of the mice fed the control diet showed none or less bioluminescent signal (Fig. 1d). To confirm that the visualized Saa3 promoter activity or the bioluminescent signal was specifically generated from the injured kidneys, we opened up the belly of Saa3 promoter-luc mice that were fed the adenine diet or the control diet and subjected them to the *in vivo* bioluminescent analysis. As shown in Fig. 1e, a very intense bioluminescent signal was specifically observed from the adenine-induced injured kidney (the white arrow), and not from the adjacent organs, whereas the uninjured kidneys and the other organs of the mice that were fed the control diet showed no bioluminescent signal. This *in vivo* result is consistent with the *ex vivo* result demonstrating that the adenine-induced injured kidney tissue exhibited a higher Saa3 promoter activity (luciferase activity) than the non-adenine-induced kidney tissue (3.5-fold, *p* < 0.01, Fig. 1f). In addition, upon adenine induction, kidney tissue showed a higher Saa3 promoter activity than other organ tissues (Supplementary Fig. S1b). These results indicated that the bioluminescent signal observed in Saa3 promoter-luc mice specifically reflected kidney injury. To assess the correlation of Saa3 promoter activity with mRNA expressions of Saa3, a fibrotic marker (collagen I (ColI)), and an inflammatory marker (TNF-α) in the injured kidneys from the tissue lysate was subjected to qualitative RT-PCR analysis and Pearson’s correlation coefficient analysis. Saa3 mRNA expression levels in the injured kidneys actually exhibited a positive correlation with Saa3 promoter activity (*r* = 0.729, *p* < 0.05, Fig. 1g). Importantly, mRNA expression levels of ColI and TNF-α were positively correlated with Saa3 promoter activity (*r* = 0.997, *p* < 0.05; *r* = 0.991, *p* < 0.05, respectively; Supplementary Fig. S3a,b). In addition to these, a significant correlation between Saa3 promoter activity with transforming growth factor beta 1 (TGF-β1) (*r* = 0.980, *p* < 0.05, Supplementary Fig. S3c) was found. These results suggested that an increase in Saa3 promoter activity was possibly associated with tubulointerstitial fibrosis and inflammation occurring in adenine-induced kidney disease. In this study, one week of the adenine diet did not change BUN and plasma creatinine concentration (Supplementary Fig. S4), and deposition of DHA crystals was not observed in renal tubules (Supplementary Fig. S2a). However, replacement of connective tissue began after only one week of the adenine diet (Supplementary Fig. S2b). Taken together, these results indicated a potential ability of the *in vivo* bioluminescence imaging from Saa3 promoter-luc mice to predict tubulointerstitial fibrosis at an early phase.

To verify the reliability of this Saa3 promoter-luc mouse model as a tool for monitoring kidney injury and tubulointerstitial fibrosis, we applied this model to assess the therapeutic effect of allopurinol, a xanthine dehydrogenase inhibitor as a positive control drug^26^, on adenine-induced kidney injury. As shown in Fig. 1h, there was no bioluminescent signal in the control group, reflecting uninjured kidney, but a strong signal intensity in the adenine group, indicating severely injured kidneys, and a decrease in the signal intensity in the adenine group treated with allopurinol, reflecting less severe injury of the kidney, due to the therapeutic effect of allopurinol. These *in vivo* bioluminescent results were confirmed by biochemistry results, in which plasma BUN and creatinine concentrations in the adenine group were significantly increased as compared to the control group but were significantly decreased by allopurinol treatment (Fig. 1i,j). Taken together, the Saa3 promoter-luc mouse model established in this study is a non-invasive and useful tool for visualizing progressive kidney injury and possibly predicting tubulointerstitial fibrosis in the early stage in live animals.

### Upregulation of Saa3 promoter activity in a unilateral ureteral obstruction model

**Figure 2:**
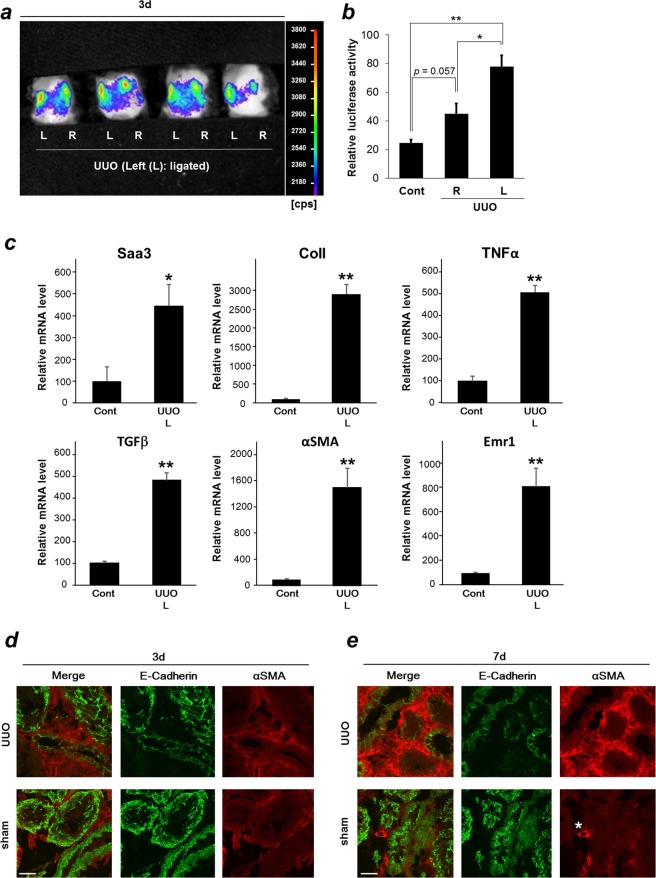
Bioluminescence imaging allows early prediction of tubulointerstitial injury and fibrosis in Saa3 promoter-luc mice under unilateral ureteral obstruction. (**a**–**c**) Saa3 promoter-luc mice were under unilateral ureteral obstruction (UUO) for 3 days (*n* = 4). Left ureters of the mice were ligated for three days (“L” represents left side and ligated kidneys), while right ureters were left without the ligation (“R” represents right side). (**a**) The *in vivo* bioluminescence imaging showed a stronger intensity of bioluminescent signal in the ligated kidneys (L) than in the unligated kidneys (R). (**b**) Kidney tissues were isolated and subjected to the *ex vivo* luciferase activity assay to show upregulation of Saa3 promoter activity in the ligated kidneys (L), as compared with the unligated kidneys (R) and control kidneys without the operation (cont) (*n* = 4). (**c**) mRNA levels of Saa3, ColI, TNF-α, TGFβ, αSMA and Emr1 in left kidney tissues of the sham (Cont) and UUO mice (UUO L) were analyzed by quantitative RT-PCR and normalized to L19 mRNA level (*n* = 5). (**d**,**e**) Wild-type mice were divided into two groups with sham operation or UUO operation. Mice were sacrificed at three or seven days after the operation. (**d**) and (**e**) Immunohistochemical staining shows expressions of E-Cad and αSMA in sham and UUO kidneys after three and seven days of the operation, respectively. αSMA protein is also expressed in the blood vessel and shown as a positive control by a star. *Scale bar*, 25 μm. All values are expressed as mean ± S.E. Student’s *t*-test, *p < 0.05, **p < 0.01.

To confirm if the Saa3 promoter-luc mouse model can be used to detect acute tubulointerstitial injury and predict tubulointerstitial fibrosis, we applied unilateral ureteral obstruction (UUO), that is an acute and well-established model of tubulointerstitial injury/fibrosis, in this study^5^. Saa3 promoter-luc mice were operated, and their left kidneys were ligated to obstruct the flow of urine, while their right kidneys were left unligated. Shortly after 3 days of the UUO operation, we subjected the mice to *in vivo* bioluminescent analysis and found that the ligated kidneys (left side indicated as “L” in Fig. 2a) showed stronger intensity of the bioluminescent signal than the unligated kidneys (right side indicated as “R” in Fig. 2a), while Saa3 promoter-luc mice with sham operation exhibited no bioluminescent signal in both the kidneys (Supplementary Fig. S5). These results suggested that acute tubulointerstitial injury, specifically upregulated Saa3 promoter activity, and higher Saa3 promoter activity visualized in ligated kidneys indicated greater severity of tubulointerstitial injury than that in unligated kidneys and sham-operated kidneys. To confirm this, kidney tissues were isolated, lysed, and subjected to the *ex vivo* quantitative luciferase assay. As compared with the basal Saa3 promoter activity from uninjured kidneys (cont), Saa3 promoter activity was increased in parallel with greater severity of tubulointerstitial injury in unligated kidneys (R) and ligated kidneys (L), respectively (Fig. 2b). Notably, the bioluminescent signal and Saa3 promoter activity observed in unligated kidneys (R) shown in Fig. 2a,b is possibly from hyperfiltration of unligated kidneys, due to the ligation of the left kidney. Subsequently, the hyperfiltration of unligated kidneys might result in intratubular hydrodynamic forces triggering inflammatory and fibrogenic signals^5^, which, in turn, caused upregulation of Saa3 promoter activity. In fact, the unligated right kidneys showed higher mRNA expression level of ColI, αSMA, and TGFβ compared with kidneys from control mice (Supplementary Fig. S6). Next, we further assessed mRNA expressions of Saa3, fibrotic markers, and inflammatory markers by subjecting the tissues of sham-operated and ligated or UUO kidneys after three days of the operation to qRT-PCR analyses and found that increased mRNA expressions of Saa3, ColI, αSMA, TGF-β, TNF-α, and EGF-like module containing mucin-like hormone receptor1 (Emr1) were clearly seen in UUO kidneys than in sham-operated kidneys (Fig. 2c). Immunohistochemical analyses revealed that severe tubulointerstitial damage was present after seven days of the UUO operation (Fig. 2e), as reflected by a highly increased αSMA expression and reduction of E-Cad expression compared with those from three day UUO kidneys (Fig. 2d). However, Saa3 promoter activity as well as mRNA expression of Saa3, ColI, αSMA, and TNF-α was upregulated early in tubulointerstitial injury (three days). In the UUO kidneys after three days of the operation, dilated tubules were observed and αSMA-positive area was moderately increased (Supplementary Fig. S7). These observations suggest that the Saa3 promoter-luc mouse model can be used to detect tubulointerstitial injury and predict fibrosis in the early stage.

### C/EBPβ plays a crucial role in controlling Saa3 promoter activity

**Figure 3:**
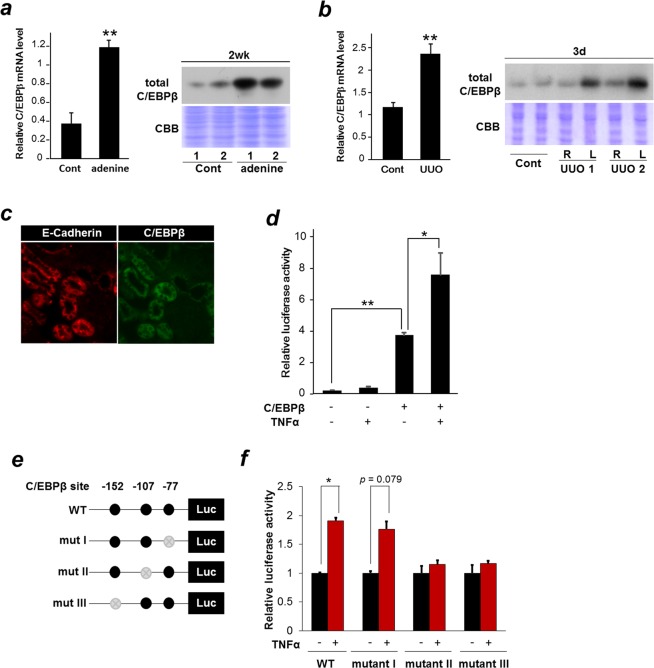
C/EBPβ is involved in regulating Saa3 promoter activity responsible for tubulointerstitial injury and fibrosis. (**a**) Wild-type (WT) mice were divided into two groups (*n* = 6) receiving the control diet (cont) or the adenine diet (adenine) for two weeks. The relative C/EBPβ mRNA expression level in kidney tissue was determined by quantitative PCR and normalized to L19 mRNA level (n = 4). Then, kidney tissues were isolated, lysed, and subjected to western blot analysis to determine C/EBPβ protein level (*n* = 2). (**b**) Left ureters of WT mice in the unilateral ureteral obstruction (UUO) group were ligated for three days (“L” represents left side), while right ureters were left without the ligation (“R” represents right side). The control group without the operation was used to compare with the UUO group. The relative C/EBPβ mRNA expression level in kidney tissue was determined by quantitative PCR and normalized to L19 mRNA level (n = 5). Then, kidney tissues were isolated, lysed, and subjected to western blot analysis to determine CEBP/β protein level (*n* = 2). (**c**) Immunohistochemical staining shows expressions of E-Cad and CEBP/β in kidney tissues from WT mice under the adenine diet for two weeks. (**d**) HK-2 cells were transiently transfected with constructs for Saa3-luc with pcDNA-C/EBPβ or empty vector. Two days after transfection, cells were treated with 10 ng/ml of TNF-α for 8 h, scraped and subjected to the luciferase assay. Data are means of triplicate experiments (*n* = 4). (**e**) Saa3 promoter was mutated and ligated upstream of the complete luciferase cDNA, generating mutant Saa3 promoter-luciferase chimeric genes (mut I, mut II, and mut III), respectively. (**f**) HK-2 cells were treated with 10 ng/ml of TNF-α for 8 h, followed by the luciferase assay. The data are from a single experiment carried out (*n* = 3) and are representative of two independent experiments. Full-length gels and blots are included in Supplementary Fig. S11.

In this study, we aimed to determine a possible role of the transcription factor, C/EBPβ in kidney injury and fibrosis by monitoring Saa3 promoter activity. First, we examined if C/EBPβ is upregulated in injured kidneys by subjecting the tissue lysates of the adenine- and UUO-treated kidneys to qRT-PCR and western blot analyses. As shown in Fig. 3a,b, C/EBPβ mRNA and protein expressions were highly increased in both adenine-treated and ligated kidneys (L) compared with uninjured kidneys in the control group (cont) and unligated kidneys (R). Taken together with the results demonstrating an upregulation of Saa3 mRNA expression and Saa3 promoter activity in the adenine- and UUO-treated kidneys (Figs 1 and 2), these results suggested a possible relationship between C/EBPβ and Saa3 expression in kidney injury. To determine which cells are responsible for C/EBPβ expression in injured kidneys, serial sections of the adenine-treated kidneys were analyzed by immunohistochemical analysis. As shown in Fig. 3c, C/EBPβ localized specifically in tubular epithelial cells, confirmed by having the same localization with E-Cad, a specific surrogate marker of renal tubular epithelial cells^27^. Given this, we carried out experiments using human renal tubular epithelial cell line HK-2 to confirm direct regulation of the Saa3 promoter activity by C/EBPβ. Upon transfection into HK-2 cells carrying Saa3 promoter-luc gene, overexpression of C/EBPβ remarkably increased Saa3 promoter activity, which was further enhanced by the addition of TNF-α, an inflammatory stimulus reported to activate C/EBPβ^12^ (Fig. 3d). C/EBPβ-biding sites are located at −152, −107, and −77 bp upstream of the transcription start site in the Saa3 promoter (Fig. 3e)^12,13,16^. Thus, we constructed three Saa3 promoter mutants at those three binding sites and the wild-type (WT) Saa3 promoter (Fig. 3e) to determine whether these C/EBPβ-binding sites are important for Saa3 promoter regulation. After transfection into HK-2 cells, both the WT and mutant construct I (−77) exhibited a 2-fold increase in luciferase activity in response to TNF-α, while mutant constructs II (−107) and III (−152) abolished luciferase activity upon TNF-α treatment (Fig. 3f). These results strongly indicated that C/EBPβ played a crucial role in regulation of Saa3 promoter activity, specifically through the C/EBPβ-biding sites at −107 and −152 bp of the Saa3 promoter. To confirm this *in vivo*, we analyzed the correlation between Saa3 promoter activity and C/EBPβ mRNA level in the adenine-treated kidney tissue by using Pearson’s correlation coefficient analysis and found that there was a significant correlation between Saa3 promoter activity and C/EBPβ mRNA level (*r* = 0.997, *p* < 0.05, Supplementary Fig. S8). In addition, C/EBPβ mRNA level was found to be significantly correlated with ColI mRNA level (*r* = 0.979, *p* < 0.05, Supplementary Fig. S8), suggesting a possible role of C/EBPβ in renal fibrosis. Taken with the previous results, the findings implied that Saa3 promoter-luc mouse model could be used to reflect C/EBPβ activity in driving tubulointerstitial injury and fibrosis. To explore the biological significance of Saa3 expression in tubular epithelial cells, we used the siRNA-mediated the human homolog serum amyloid A (SAA1) knockdown in HK-2 cells. The amount of reduced mRNA expression was assessed by quantitative RT-PCR (Supplementary Fig. S9). Silencing of the SAA1 RNAi gene resulted in a decreased interleukin-8 (IL-8) mRNA expression in HK-2 cells (Supplementary Fig. S9). Because renal tubular epithelial cells are studied to produce inflammatory mediators, such as cytokines and chemokines, and actively participate in acute inflammatory processes by effecting and directing leukocyte chemotaxis via the production of IL-8^28^, these data suggest that SAA1 is actually involved in the early pathological process of kidney fibrosis and SAA1 serum levels might be useful assessing early stage human renal disease.

### Application of Saa3 promoter-luc mouse model in searching for functional food preventing kidney disease

**Figure 4:**
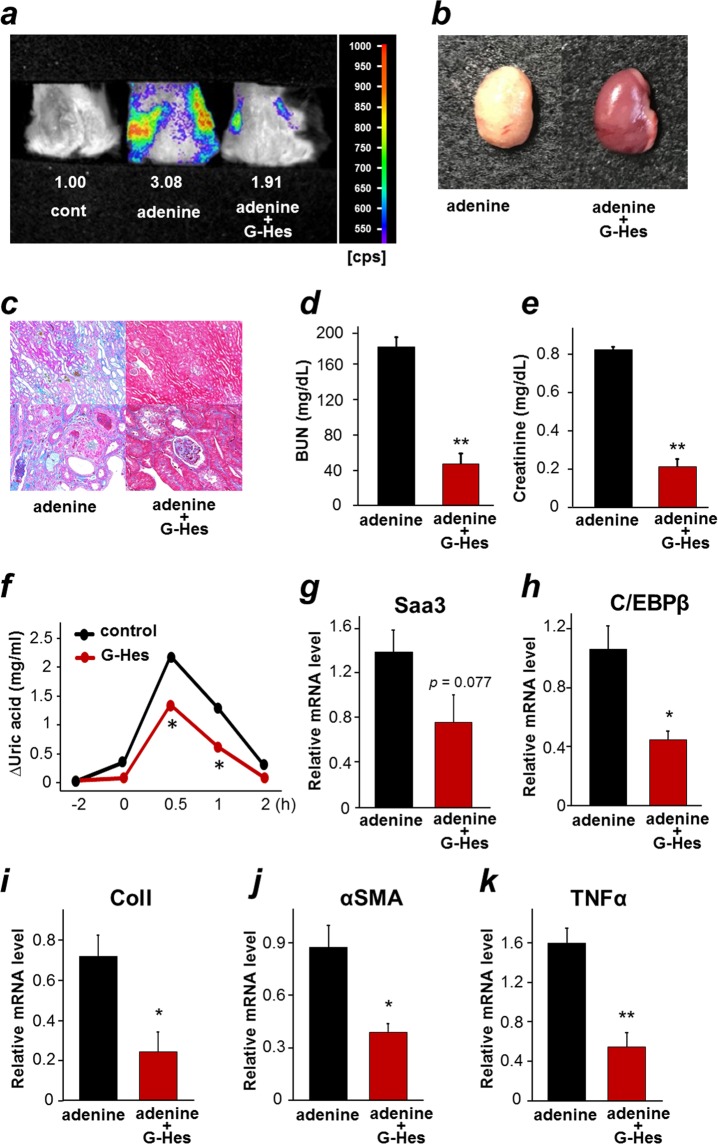
Bioluminescence imaging of Saa3 promoter-luc mice allows monitoring the response to glucosyl-hesperidin therapy in adenine-induced kidney disease model. (**a**) The treatment of glucosyl-hesperidin (G-Hes) given to Saa3 promoter-luc mice receiving the adenine diet for one week. The *in vivo* bioluminescence imaging shows decreased signal intensity in the adenine-induced mouse treated with G-Hes, reflecting less severe injury in kidney, as compared with the adenine-induced mouse without G-Hes treatment. (**b**–**e**) Wild-type mice were divided into two groups (*n* = 6) receiving the adenine diet (adenine) or the G-Hes-mixed adenine diet (adenine + G-Hes) for three weeks. (**b**) Appearance of kidneys in both groups. (**c**) Kidneys were sectioned and stained with Azan-Mallory to show morphological changes. (**d**) Plasma BUN and (**e**) creatinine levels were suppressed by the G-Hes treatment (*n* = 6). (**f**) G-Hes treatment reduced plasma uric acid levels, exhibiting the inhibition of endogenous xanthine oxidoreductase (XOR) activity, (*n* = 5). (**g**–**k**) The relative mRNA level of Saa3, CEBP/β, ColI, αSMA, and TNF-α, respectively, in kidney tissues of mice from the experiment in (**b**–**e**)was determined by quantitative PCR and normalized to L19 mRNA level (*n* = 6). All values are expressed as mean ± S.E. Student’s *t*-test, *p < 0.05, **p < 0.01.

Citrus flavonoids have been reported to have protective kidney functions, including prevention of renal crystal formation^29^. In this study, we decided to use Saa3 promoter-luc mouse model for monitoring the potential preventive effect of citrus flavonoids on adenine-induced kidney disease. Since preliminary studies with certain types of flavonoids revealed a possibility of glucosyl-hesperidin (G-Hes) in preventing kidney injury, we subjected G-Hes to the *in vivo* bioluminescence imaging model. Transgenic mice were divided into three groups: the control group receiving the control diet, the adenine group receiving the adenine diet, and the G-Hes receiving the G-Hes-mixed adenine diet for one week. Three days prior to the treatment, the G-Hes group received the G-Hes diet, while the control and adenine groups received the control diet. After one week of the treatment, we subjected all mice to *in vivo* bioluminescent analysis. As shown in Fig. 4a, *in vivo* bioluminescence imaging showed strong signal intensity in the adenine group, indicating severely injured kidneys, and a decrease in the signal intensity in the G-Hes group (Supplementary Fig. S10a), indicating less severe injury of the kidneys, possibly due to the therapeutic effect of G-Hes, while no bioluminescent signal was observed in uninjured kidney of the control group (cont) that confirmed the specificity of Saa3 promoter activity to the injury. This result strongly suggested a preventive effect of G-Hes on adenine-induced kidney disease. To further confirm the preventive effect of G-Hes, after 3 weeks of the above treatment, all mice were sacrificed and subjected to further analyses. Figure 4b shows abnormal, ivory-white appearance of the adenine-treated kidney possibly due to DHA crystal accumulation, while the G-Hes treatment group showed usual appearance of kidney, suggesting the possibility of G-Hes in preventing DHA crystal formation. To observe morphological changes, the kidneys were sectioned and stained with Azan-Mallory stain. It appeared that G-Hes suppressed deposition of DHA crystals in renal tubules (brown), expansion of ECM, tubular dilation and atrophy, and tubulointerstitial fibrosis (blue), which were greatly present in the adenine group (Fig. 4c and Supplementary Fig. S10b,c). Consistent with histochemical staining results, G-Hes showed renal-protective effects by the significant reduction of plasma BUN and creatinine concentrations (Fig. 4d,e). Since G-Hes showed a potential suppression of DHA crystal formation in kidneys, we were interested in investigating the inhibitory effect of dietary G-Hes on activity of xanthine oxidoreductase (XOR), an enzyme responsible for conversion of adenine to DHA^26^. As shown in Fig. 4f, G-Hes suppressed XOR activity *in vivo*, indicated by the significant reduction of plasma levels of uric acid, a metabolic product of XOR, suggesting a possible renal protective effect of G-Hes through suppression of DHA crystal formation. To ensure that a reduction in plasma uric acid levels was due to the decreased activity of XOR by G-Hes and not by uricase, the uricase inhibitor, potassium oxonate, was applied during XOR activity assessment. Given that G-Hes is hydrolyzed to hesperetin by gut flora prior to being absorbed, we further confirmed the inhibitory effect of hesperetin on *in vitro* XOR activity from milk (IC_50_ value was 10.6 μM). Qualitative RT-PCR analysis revealed that G-Hes treatment downregulated mRNA expressions of Saa3, C/EBPβ, ColI, αSMA, and TNF-α (Fig. 4g–k), indicating a possible role of G-Hes in suppressing inflammation and fibrosis in adenine-induced kidney disease. Taken together, the results suggested that G-Hes exerts its renal preventive effect through suppression of accumulation of DHA crystals in tubules via inhibition of XOR activity, thereby preventing tubular injury subsequent to reduction of inflammation, leading to prevention of further tubular injury and fibrosis in the kidneys. Notably, by exploiting the specificity of Saa3-promoter activity, this preventive effect could be visualized by bioluminescence imaging of live mice.

## Discussion

Scientific testing on animals has become a moral and social point of contention. In recent years, efforts have been made to reduce the number of experimental animals and to establish new non-invasive methods. *In vivo* bioluminescence imaging as a potent non-invasive assessment has been applied in various disease models, such as inflammation diseases and cancer metastasis^11,12,30–32^. However, until this point, this technology has not been utilized in kidney disease research. Examination by non-invasive imaging for renal fibrosis in both human and experimental animals, in particular, is nonexistent. Generally, tubulointerstitial fibrosis, a predictor of the progression of end-stage renal disease^33^, in patients is commonly assessed by biopsy and histological analysis^5,6,33^. Similarly, in experimental animals, analysis of tubulointerstitial fibrosis is often done by histochemical and/or gene and protein expression analyses, which require tissue or organ harvesting after sacrificing the animal; therefore, disease progression or therapeutic response in the same animal over time cannot be observed. To overcome these limitations, here we established and demonstrated that *in vivo* Saa3/C/EBPβ-promoter bioluminescence imaging is a sensitive and specific tool for detecting and visualizing tubulointerstitial injury in real time in live animals from both primary kidney disease models, adenine-derived, and UUO models. Moreover, this novel bioluminescence imaging has an advantage of early prediction of tubulointerstitial fibrosis, which cannot be detected or clearly distinguished by the typical and traditional histological analysis or blood markers such as BUN and creatinine at an early phase.

C/EBPβ is a transcription factor of various genes including Saa, αSMA, and Col I^12,13,17,18,34–36^. C/EBPβ regulates expression of its target genes by binding to its binding sites in the promoter region of these genes subsequent to gene transcription. Recently, C/EBPβ has been reported to play a crucial role in chronic inflammatory diseases, such as rheumatoid arthritis^13,16^ and obesity^12^. Based on these findings, researchers have designed lentiviruses carrying Saa3/C/EBPβ-promoter reporter gene and used them as a sensitive tool for assessing arthritis and chronic inflammatory state in experimental animals^12,13^. In addition to involvement in chronic inflammatory diseases, recent emerging evidence has suggested a crucial rule of C/EBPβ in fibrosis^17,18,35,36^. In lung and liver fibrosis, its mechanism of action works by controlling transcription of αSMA and ColI genes and regulating fibroblast activation and myofibroblast differentiation via the TGF-β pathway. C/EBPβ-deleted mice exhibited a decrease in αSMA and ColI mRNA and protein levels and suppression of TGF-β-induced fibroblast differentiation into myofibroblasts subsequent to attenuation of ECM accumulation and fibrosis^17,18,35–37^. In renal injury and disease, there is a consensus that the transformation of fibroblasts to myofibroblasts plays a pathological role in interstitial ECM accumulation during progressive tubulointerstitial fibrosis as assessed by upregulated expression of αSMA and other related ECM proteins^5,6,33,38^. Although the origin of interstitial myofibroblasts during renal fibrosis is controversial, TGF-β1 is a key mediator activating various cells, such as tubular epithelial cells, resident fibroblasts, and bone marrow–derived mesenchymal stem cells whereby they lose their normal phenotypes and acquire a myofibroblast phenotype, accelerating matrix synthesis in renal fibrosis^33,38,39^. In our study, we found that TGF-β1 mRNA expression level was positively correlated with Saa3 promoter activity. This suggests that the increased bioluminescent signal intensity observed in Saa3 promoter-luc mice reflected differentiation and activation of myofibroblasts in tubulointerstitial fibrosis.

In this study, Saa3 gene promoter activity was applied to predict tubulointerstitial fibrosis in both chronic and acute renal injuries. Thus, the pathological relationship between Saa3 and kidney disease and fibrosis should be discussed. Recent studies coincidentally reported the upregulation of members of the Saa family in plasma and kidney tissues of patients and experimental animals having kidney disease^20–25^. Not solely in the kidney, Saa was also found to play a role in fibrosis in other organs, including the lung, pancreas, and liver, in which Saa3-deficient mice exhibited markedly reduced ECM and fibrosis and a positive correlation between Saa expression with fibrotic markers and fibrotic severity was observed^20,40,41^. Although molecular mechanisms of Saa in fibrosis development have been unclear, recent studies suggest its possible association with inflammatory-related pathways. Saa3 has been reported to be an endogenous ligand for toll-like receptor 4 (TLR4), a receptor recently found to be implicated in inflammation and fibrosis^42,43^. Thus, it is possible that Saa3 may exert its fibrotic function through activation of the TLR4 signaling pathway. In response to injury or inflammation, Saa is highly synthesized in local tissue^21,44–46^. In diabetic kidney disease (DKD), Saa was found to be expressed throughout the kidney and highly deposited in tubulointerstitial fibrotic areas in kidney tissues of both the patients and experimental animals^21^. Under diabetes-like conditions (exposure to advanced glycation products (AGE)), Saa and inflammatory mediators, such as C-X-C motif chemokine ligand 5 (Cxcl5), C-C motif chemokine ligand 2 (Ccl2), and C-C motif chemokine ligand 5 (Ccl5) were significantly upregulated in renal podocytes^47^. Notably, knocking out Saa3 significantly inhibited AGE-induced expressions of Cxcl5, Ccl2, and Ccl5 in podocytes, while exogenous addition of recombinant Saa protein upregulated Saa3 itself and inflammatory mediators associated with nuclear factor-κB (NF-κB) activation^21,47^. Taken together with the notion that Cxcl5, Ccl2, and Ccl5 are chemoattractants, these results indicate that Saa acted in a chemoattractive manner to modulate immune cell recruitment in injured or inflammatory sites in the kidney. Interestingly, some studies demonstrated that Saa was a connector binding ECM and immune cells, such as neutrophils, monocytes, T cells, and mast cells, providing cross talk between these cells and the accumulation of immune cells in inflammatory sites^44,48^. In the kidney, accumulation of immune cells and Saa in parallel with upregulation of TGF-β1 and αSMA were found in interstitial fibrotic areas, leading to an assumption that the presence of immune cells and Saa enhanced renal fibrosis^44^. More recent works examined an important role of macrophage recruitment during the development of renal fibrosis^49,50^. It showed that the recruited macrophages were mainly M1 macrophages at early stage (three days after the UUO operation) and then were shortly polarized into M2 macrophages, which released high levels of TGF-β1 to promote renal fibrosis^50^. This work is consistent with previous studies suggesting that TGF-β1 was a key mediator, controlling the phenotypic transition of resident renal cells into myofibroblasts, leading to fibrosis^33,38,39^. Although these observations suggest the involvement of Saa3 in the renal fibrotic process via inflammatory-related pathways, further experiments are needed to clarify the causal relationship between bioluminescent signal visualized in Saa3 promoter-luc mice and immune cell recruitment.

Finally, we applied Saa3 promoter-luc mice for the *in vivo* non-invasive exploration of food factors preventing kidney disease in the adenine-induced kidney disease model. We found that G-Hes effectively prevented renal inflammation and fibrosis, which accompanied inhibition of DHA crystal deposition in renal tubules. We also found the inhibitory effect of its hydrolyzed form, hesperetin, on XOR activity *in vitro*. As XOR is a critical enzyme, catalyzing the oxidation of hypoxanthine to uric acid^51^, we can expect that G-Hes would be a food factor preventing hyperuricemia with excess uric acid in the blood. This study provides a novel approach to investigate effects of functional food by visualizing bioluminescent signal in Saa3 promoter-luc mice. As described above, Saa3 is highly expressed in tubulointerstitial fibrotic areas in kidney tissues of mouse DKD models, whereas previous reports have demonstrated that Saa3 facilitated pulmonary metastasis through stimulating NF-κB/TLR4 signaling pathways^43,52^. Taken together with these observations, Saa3 promoter-luc mouse model can be widely applied as an *in vivo* non-invasive model in exploring compounds or food factors for preventing not only DKD but also other diseases, such as cancer metastasis.

## Materials and Methods

### Establishment of Saa3 promoter-luc chimeric mice

Saa3/C/EBPβ-promoter transgenic mouse carrying mouse Saa3 promoter region (−314/+50) upstream of the complete luciferase cDNA was generated as described previously^12,13,16^. In this experiment, heterozygous mice harboring the transgene which were backcrossed at least five times with purebred C57BL/6J mice (Charles River Japan, Kanagawa, Japan), and their control littermates were used. Male CD-1 (ICR) mice (four weeks old) were from Charles River Japan. Mice were housed in groups of two or three in metal cages maintained at 24 °C with a 12-h light and dark cycle (lights on, 8:00 a.m. to 8:00 p.m.). MF solid chow (Oriental Yeast, Tokyo, Japan) and deionized water were provided *ad libitum*. The animal study was approved by the Hiroshima University Animal Committee (Permit Number: C13-3 and C18-2), and the mice were maintained in accordance with the Hiroshima University Guidelines for the Care and Use of Laboratory Animals.

### Adenine-induced and UUO kidney injury experiments

Male C57BL/6J mice (eight weeks old, Charles River Japan) as WT mice and Saa3 promoter-luc mice (5-8 weeks old, established in this study) were housed in groups of two or three in metal cages under the conditions described above. The control diet used in all experiments was composed of the following components (g/kg diet): α-cornstarch, 402; casein, 200; sucrose, 200; corn oil, 100; cellulose, 50; AIN-93G mineral mixture, 35; AIN-93 vitamin mixture, 10; and L-cystine, 3, as described previously^53^.

For the adenine-induced kidney injury experiment, the adenine diet was composed of the control diet mixed with adenine (FUJIFILM Wako, Osaka, Japan) in a dose of 2 g adenine/kg diet (0.2% w/w). This adenine dose was chosen because it showed a survival rate of 100% with the renal injury phenotypes reported previously^26^. The G-Hes diet was composed of the control diet mixed with G-Hes (Hayashibara, Okayama, Japan) (3% w/w), while the G-Hes-mixed adenine diet was composed of the adenine diet mixed with G-Hes (3% w/w). Both WT mice and Saa3 promoter-luc mice were randomly divided into the control group receiving the control diet, the adenine group receiving the adenine diet, and the G-Hes group receiving G-Hes-mixed adenine diet for one, two, or three weeks. In the G-Hes group, mice were fed the G-Hes diet for 3 days before the start of the adenine treatment. For allopurinol experiment, the allopurinol group was given allopurinol (25 mg/kg/day; Sigma, St. Louis, MO) in the drinking water from 2 weeks after the start of the adenine treatment until end of the experiment (six weeks). At the end of each experiment, mice were sacrificed, and organs and blood were harvested. The *n* for each experimental group is defined within the figure legends.

For the UUO kidney injury experiment, WT mice and Saa3 promoter-luc mice were randomly divided into the UUO group or the sham group, and their left ureters were ligated with 6-G silk sutures, as previously described^50,54^. For sham operation, mice were treated the same way as UUO mice, except the ligation. After surgery, mice were fed the control diet and then sacrificed at three or seven days as mentioned in the legends of figures.

### Plasmid construction, cell culture, and DNA transfection

Mouse Saa3 promoter region (−314/+50) was ligated upstream of the luciferase cDNA in pGEM3 (Promega, Madison, WI, USA), generating pSaa3-Luc. pSaa3-Luc was digested with both *Eco*RI and *Not*I, and the resulting DNA fragment containing the mouse Saa3 promoter region (−314/+50) and luciferase cDNA (Saa3-Luc) was ligated into *Eco*RI and *Not*I sites of the pMX vector. Site-directed mutagenesis was performed using PrimeSTAR HS DNA Polymerase (TaKaRa Bio) as described previously^12^. The mutation was confirmed by DNA sequencing analysis. C/EBPβ cDNA was amplified using a PCR primer set (5′-CCGCGTTCATGCACCGCCTG-3′ and 5′-ACCCGCGCCGCGCTAGCAGT-3′) and subcloned into pcDNA3.1, which generated pcDNA-C/EBPβ. The human proximal tubular cell line HK-2 was cultured in a maintenance medium (10% fetal bovine serum, 100 units/ml penicillin and 100 μg/ml streptomycin in Dulbecco’s modified medium with low glucose) at 37 °C in 5% CO_2_/95% humidified air. DNA transfections were performed using GeneJuice Transfection Reagent (Merck Millipore, Billerica, MA) according to the manufacturer’s instructions. After DNA transfection into HK-2 cells, these cells were stimulated with 10 ng/ml of mouse recombinant tumor necrosis factor-α (R&D system, Minneapolis, MN) for 24 h.

### *In vivo* bioluminescence imaging analysis

Male Saa3 promoter-luc mice were injected intraperitoneally with D-luciferin (150 mg/kg body weight, Promega) and then anesthetized with pentobarbital sodium (Kyoritsu Seiyaku, Tokyo, Japan). After five min, Saa3-luc mice were placed in a prone position on the plate and imaged for 1 min with the camera set at the highest sensitivity level by NightOWL II Imaging Systems LB983 (Berthold Technologies, Bad Wildbad, Germany), except the mice shown in Fig. 1e (they were placed in a supine position). Photons emitted from tissues were analyzed using Indigo *in vivo* image software (Berthold). Signal intensity was quantified as the sum of all detected photon counts per second and presented as count/sec (cps). For any given analysis, all images were adjusted to the same scale of minimum and maximum luminescent intensity.

### *Ex vivo* quantitative luciferase assay

Tissues obtained from Saa3 promoter-luc mice were homogenized in the lysis buffer (1% Triton-X 100, 2 mM DTT, 10% glycerol, and 25 mM Tris-HCl pH 7.5), followed by centrifugation at 12,000 rpm, 4 °C, for 10 min and supernatant collection. For cell lysate, the cells were lysed in ice-cold lysis buffer. After sonication, cell lysate was centrifuged at 12,000 rpm and supernatant was collected. Luciferase activity was quantified using the luciferase assay kit (Toyo Inki, Japan) and a luminometer (Turner Model TD-20), expressed as relative light units normalized to total protein concentration of the cell or tissue extracts.

### RT-PCR analyses

Total RNAs were isolated from kidney using RNeasy lipid tissue kit (Qiagen Sciences, Germantown, MD). The reverse transcriptase reaction was carried out with 1 μg total RNA as a template to synthesize cDNA using ReverTra Ace (TOYOBO, Osaka, Japan) and random hexamers (TaKaRa Bio, Kyoto, Japan), according to the manufacturer’s instructions. For semi-quantitative PCR analysis, cDNA and primers were added to the GoTaq Master Mix (Promega) to give a total reaction volume of 20 µl. Reactions were sampled after 28 and 30 cycles under different PCR conditions, to monitor product accumulation. For quantitative PCR analysis, cDNA and primers were added to the THUNDERBIRD SYBR qPCR Mix (TOYOBO), to give a total reaction volume of 20 µl. PCR reactions were then performed using StepOnePlus^TM^ (Applied Biosystems, Foster City, CA). Conditions were set to the following parameters: 2 min at 95 °C, followed by 40 cycles each of 15 s at 95 °C and 1 min at 60 °C. The primers used for PCR analyses were as follows: Saa3, F, 5′-AAGGGTCTAGAGACATGTGG-3′, and R, 5′- ACTTCTGAACAGCCTCTCTG -3′; TNF-α, F, 5′-CCGAT GGGTTGTACCTTGTC-3′, and R, 5′-CGGACTCCGCAAAGTCTAAG-3′; αSMA, F, 5′-GGCTCTGGGCTCTGTA-3′, and R, 5′-CTCTTGCTCTGGGCTTCATC-3′; Col1, F, 5′-CCCAAGGAAAAGAAGC-3′, and R, 5′-ACATTAGGCGCAGGAAGGTCA-3′; L19, F, 5′-GGCATAGGGAAGAGGAAGG-3′, and R, 5′-GGATGTGCTCCATGAGGATGC-3′; C/EBPβ, F, 5′-ACAAGCTGAGCGACGGTAC-3′, and R, 5′-ACAGCTGCTCCACCT TCTTC-3′.

### Immunohistochemical analysis

Kidney tissues were fixed in 4% paraformaldehyde. Paraffin sections of 4 µm were stained with H&E and Azan-Mallory stain. For immunofluorescence staining in Fig. 2d,e (three or seven days of UUO) and Fig. 3c (two weeks of the adenine treatment) frozen kidneys were sliced with 5-µm thickness. The slices were fixed with acetone or 4% paraformaldehyde. After blocking in 5% donkey serum for 30 min at room temperature, slices were incubated with primary antibody, followed by Alexa Fluor labeled (Molecular probe) secondary antibody. The images were captured by confocal microscopy (FV2000, Olympus) and were processed by Olympus fluoview ver.4.0. Antibodies of αSMA (Cell Signaling, 1A4, 48939), E-Cad (Abcam, DECMA-1, ab11512), and CEBP/β (c-7962; Santa Cruz Biotechnology) were used.

### Western blot analysis

Kidney tissues were homogenized in the lysis buffer (150 mM NaCl, 1% Nonidet P-40, 0.5% sodium deoxycholate, 0.1% SDS, 50 mM Tris-HCl, pH 7.4) followed by centrifugation at 12,000 rpm, 4 °C, for 10 min and supernatant collection. Protein concentration of the supernatant was determined using the Bio-Rad protein assay kit (Bio-Rad) with BSA as a standard. 20 μg (protein equivalents) of the supernatant was resolved by SDS-PAGE, transferred onto a polyvinylidene difluoride (PVDF) membranes and immunoblotted with anti-CEBP/β (Santa Cruz Biotechnology) antibody.

### Plasma BUN and creatinine analysis

BUN and creatinine were determined using a Beckman Coulter AU480 analyser (Beckman Coulter, Krefeld, Germany), which is an automated chemistry instrument for turbidimetric, spectro-photometric, and ion-selective electrode measurements. Briefly, 200 μL plasma was used to measure these parameters according to the manufacturer’s protocol.

### XOR activity analysis

WT mice were randomly divided into the control group or the G-Hes group. Before starting the experiment, the G-Hes group was treated with G-Hes (500 mg/kg body weight) in saline solution by a single oral administration for 2 h, while the control group was treated with saline solution. After 2 h, all mice were treated with the uricase inhibitor, potassium oxonate (250 mg/kg *i*.*p*., FUJIFILM Wako)^55^ in saline solution. Blood samples (100 μl each) were collected at 0, 0.5, 1, and 2 h after potassium oxonate injection by cutting the tail tip. Prior to G-Hes treatment, blood was also collected as the basal urate level. Plasma was obtained by centrifugation at 3000 rpm, for 10 min, and at 4 °C. Plasma urate levels were determined by high-performance liquid chromatography (HPLC) using a Cosmosil 5C_18_-PAQ (4.6 × 250 mm; Nacalai Tesque, Kyoto, Japan) with an isocratic elution of 20 mM phosphate buffer (pH 2.5) at a flow rate of 0.5 ml/min, a temperature of 30 °C, and a monitored wavelength of 290 nm.

### Statistical analyses

Values were presented as means ± S.E. Statistical significance was determined by unpaired Student’s t test. For all tests, the results with *p* < 0.05 were considered statistically significant.

## Supplementary information

Supplementary Figures
