# Supplementary material for: The serum amyloid A3 promoter-driven luciferase reporter mice is a valuable tool to image early renal fibrosis development and shows the therapeutic effect of glucosyl-hesperidin treatment
Source: Sci Rep. 2019 Oct 1;9:14101. doi: 10.1038/s41598-019-50685-0 (PMC6773767; doi:10.1038/s41598-019-50685-0)

The serum amyloid A3 promoter-driven luciferase reporter mice is a valuable tool to image early renal fibrosis development and shows the therapeutic effect of glucosyl-hesperidin treatment

Thanutchaporn Kumrungsee[1,*], Taishi Kariya[1], Kotaro Hashimoto[1], Takayuki Koyano[2], Nao Yazawa[1], Takao Hashimoto[1], Yohei Sanada[1], Makoto Matsuyama[2], Yusuke Sotomaru[3], Hiroaki Sakurai[4], Fons AJ van de Loo[5], and Noriyuki Yanaka[1,*]

[1]Graduate School of Biosphere Science, Hiroshima University, Higashi-Hiroshima, Japan

[2]Division of Molecular Genetics, Shigei Medical Research Institute, 2117 Yamada, Minami-ku, Okayama, Japan

[3]Natural Science Center for Basic Research and Development, Hiroshima University, Hiroshima, Japan.

[4]Department of Cancer Cell Biology, Graduate School of Medicine and Pharmaceutical Sciences, University of Toyama, Toyama, Japan

[5]Experimental Rheumatology, Department of Rheumatology, Radboud University Medical Center, Nijmegen, The Netherlands.

Supplementary figures:

Supplementary figure S1

Supplementary figure S2

Supplementary figure S3

Supplementary figure S4

Supplementary figure S5

Supplementary figure S6

Supplementary figure S7

Supplementary figure S8

Supplementary figure S9

Supplementary figure S10

Supplementary figure S11

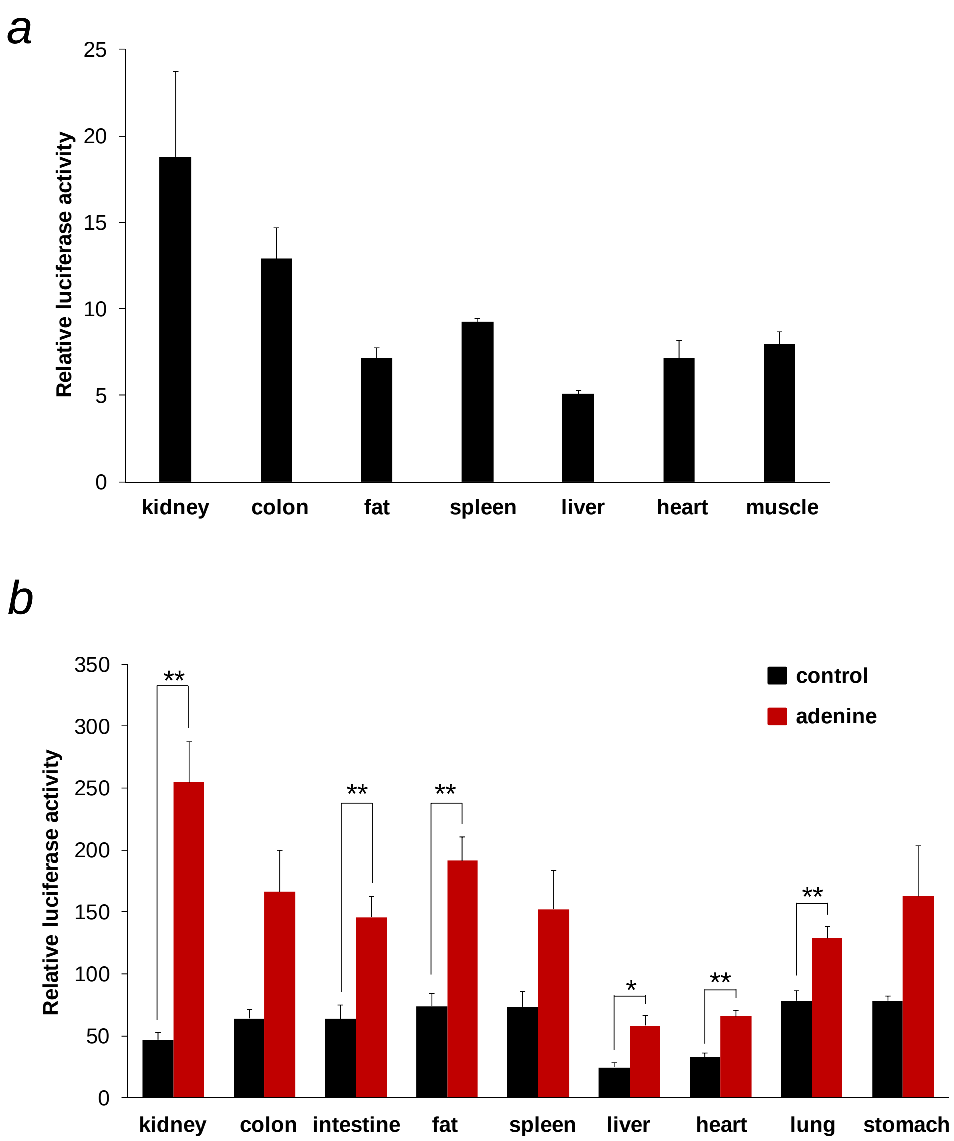

Supplementary figure S1. Saa3 promoter activity (luciferase activity) of various tissues isolated from Saa3 promoter-luc mice.

a, The transgenic mice showed high Saa3 promoter activity in kidney tissue under normal conditions (n = 3). *b*, The 1-week adenine-induced kidney tissue exhibited the highest Saa3 promoter activity (luciferase activity) among other organ tissues and higher than the non-adenine-induced kidney tissue (n = 3). All values are expressed as mean ± S.E. Student's *t*-test, $*p < 0.05$, $**p < 0.01$.

a

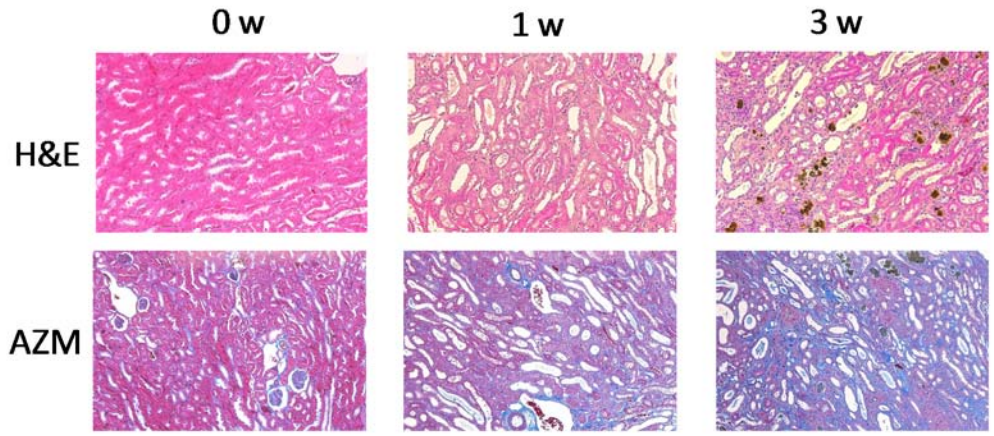

b

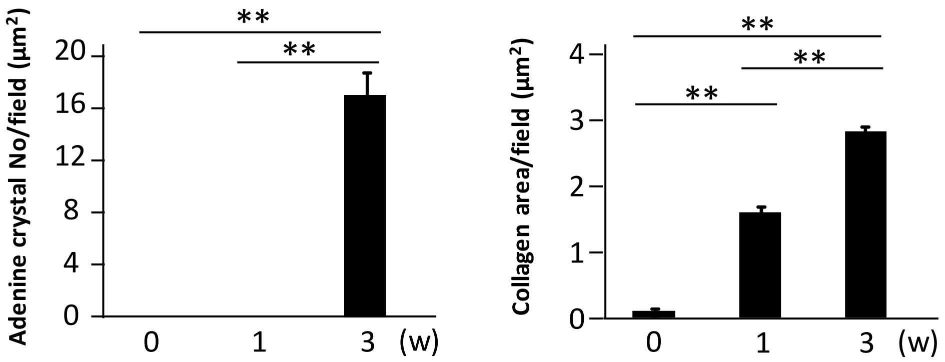

Supplementary figure S2. Histopathological image analyses of the adenine-induced kidney.

Wild-type mice were given the adenine diet for 0, 1, or 3 weeks (w). Kidney tissues were isolated at each time point and paraffin-embedded sections were subjected to hematoxylin and eosin (H&E) or Azan-Mallory (AZM) staining. At 3 weeks, deposition of 2,8-dihydroxyadenine (DHA) crystals in renal tubules (brown), expansion of interstitial extracellular matrix, and accumulation of collagen, which are typical pathologies of tubulointerstitial fibrosis, were observed. At 1 week, deposition of DHA crystals is not seen in renal tubules, however, replacement to connective tissue has already started. Statistical significance was analyzed with unpaired *t*-test, and qualitative

histopathological score was with Mann-Whitney U test. Time-course interaction with renal crystal formation and accumulation of collagen was analyzed with a two-way ANOVA without replication followed by non-parametric Steel-Dwass multiple comparison test. For all tests, the results with $p<0.05$ (2-sided) were considered statistically significant.

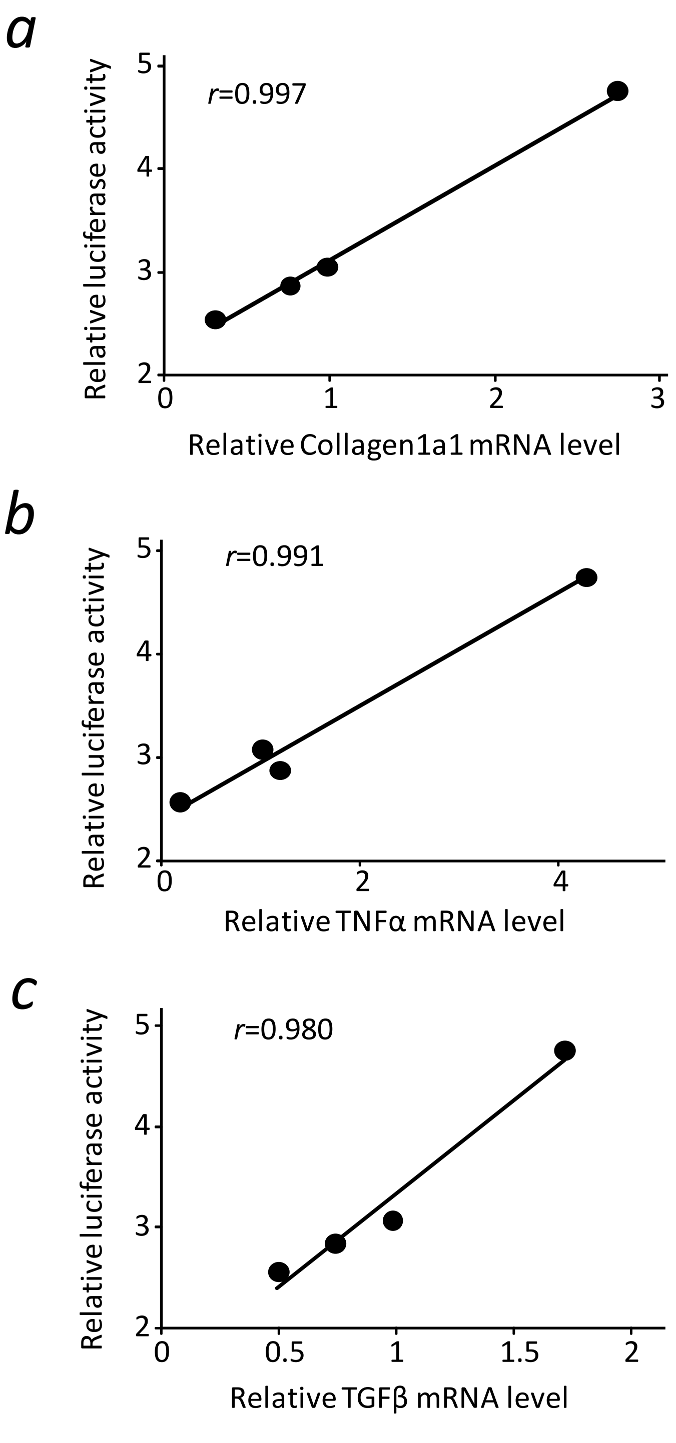

Supplementary figure S3. Positive correlation between luciferase activity and mRNA expression levels of collagen Ia1 (ColI), TNFα and TGFβ in adenine-induced kidney disease. Saa3 promoter-luc mice were given the adenine diet for 1 week. Kidney tissues

were isolated and subjected to the *ex vivo* luciferase activity assay (n = 4). The relative mRNA expression levels of ColI, TNFα, and TGFβ in kidney tissue were determined by quantitative PCR and normalized to L19 mRNA level (n = 4). Pearson's correlation coefficient showed a positive correlation between luciferase activity (promoter activity) and mRNA expression levels of ColI (*a*), TNFα (*b*) and TGFβ (*c*) in kidney tissue.

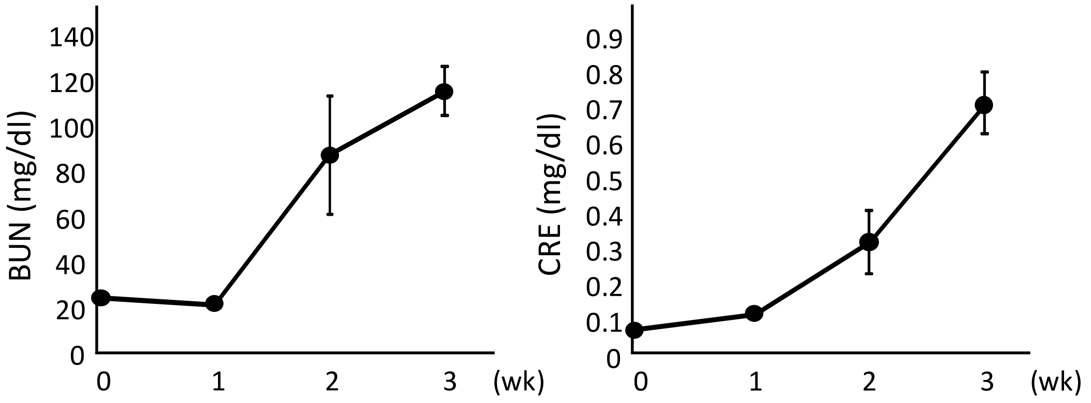

Supplementary figure S4. Blood urea nitrogen (BUN) and plasma creatinine concentrations during adenine-induced kidney disease development.

Wild-type mice were given the adenine diet for 0, 1, 2, or 3 weeks. After serum was collected by a 10-min centrifugation at 8000 rpm, BUN and creatinine were determined using a Beckman Coulter AU480 analyser (Beckman Coulter, Krefeld, Germany), which is an automated chemistry instrument for turbidimetric, spectro-photometric, and ion-selective electrode measurements. Briefly, 200 μL plasma was used to measure these parameters according to the manufacturer's protocol.

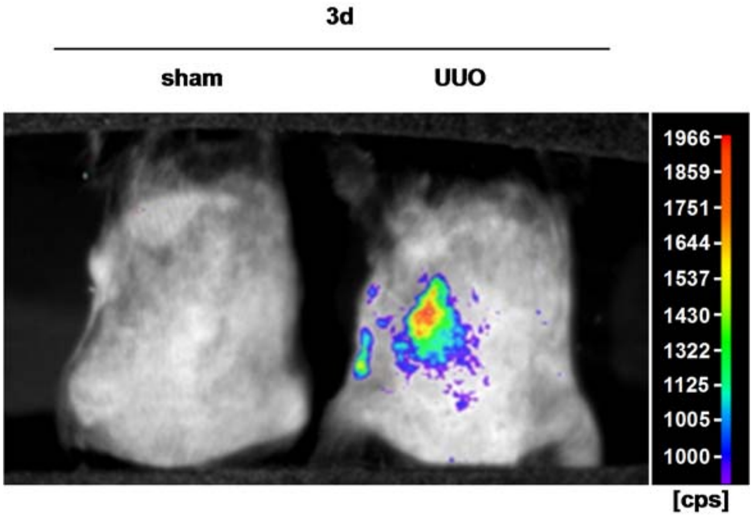

Supplementary figure S5. Bioluminescence imaging of Saa3 promoter-luc mice under sham operation or unilateral ureteral obstruction. Saa3 promoter-luc mice were divided into the UUO group or the sham group, and their left ureters were ligated. For sham operation, the mice were treated the same way as the UUO mice, except the ligation. Three days after surgery, the mice were subjected to *in vivo* bioluminescent analysis.

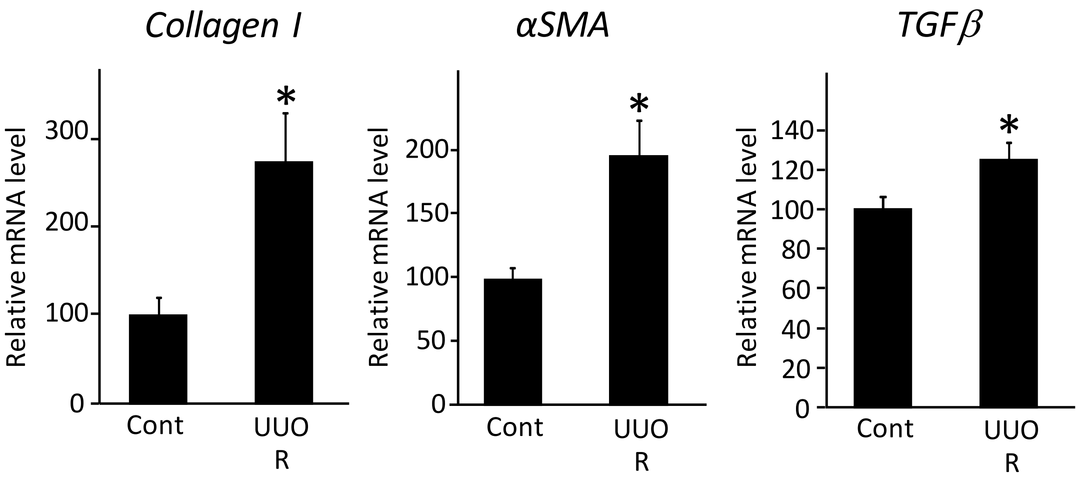

Supplementary figure S6. Response of mRNA expressions in unligated right kidneys (R) to unilateral ureteral obstruction. Wild-type mice were divided into the unilateral ureteral obstruction (UUO) group or the sham group, and their left ureters were ligated. For sham operation, the mice were treated the same way as the UUO mice, except the ligation. Three days after the surgery, unligated right kidneys were isolated and subjected to mRNA expression analyses to show upregulation of ColI, αSMA and TGFβ mRNAs in the unligated kidneys (R), as compared with control kidneys without the operation (Cont) (n = 5). The relative mRNA expression levels of ColI, αSMA and TGFβ in kidney tissue were determined by quantitative PCR and normalized to L19 mRNA level. All values are expressed as mean ± S.E. Student's *t*-test, *$p < 0.05$.

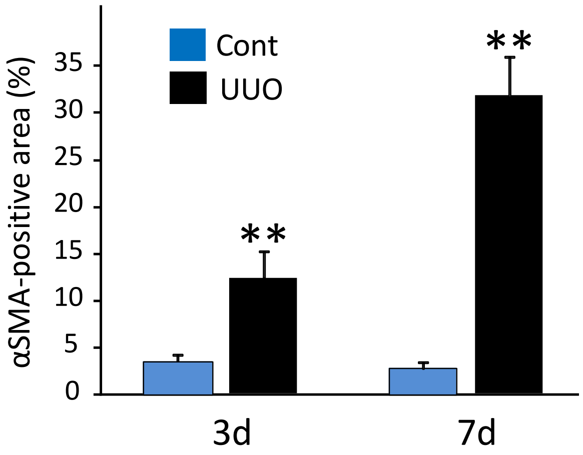

Supplementary figure S7. Increased in αSMA-positive cells in the ligated kidneys. Wild-type mice were divided into the unilateral ureteral obstruction (UUO) group or the sham group, and their left ureters were ligated. For sham operation, the mice were treated the same way as the UUO mice, except the ligation. Three days or 7 days after the surgery, left kidneys were isolated. Frozen kidneys were sliced with 5 μm thickness and fixed with acetone. After blocking in 5 % donkey serum for 30 min at room temperature, slices were incubated with anti-αSMA antibody (Cell Signaling, 1A4, 48939), followed by Alexa Fluor labeled (Molecular probe) secondary antibody. The images were captured by confocal microscopy (FV2000, Olympus) and processed by Olympus fluoview ver.4.0. αSMA positive areas were calculated using ImageJ software (National Institutes of Health) with five randomly chosen fields in each slice. All values are expressed as mean ± S.E. Student's *t*-test, $^{**}p < 0.01$.

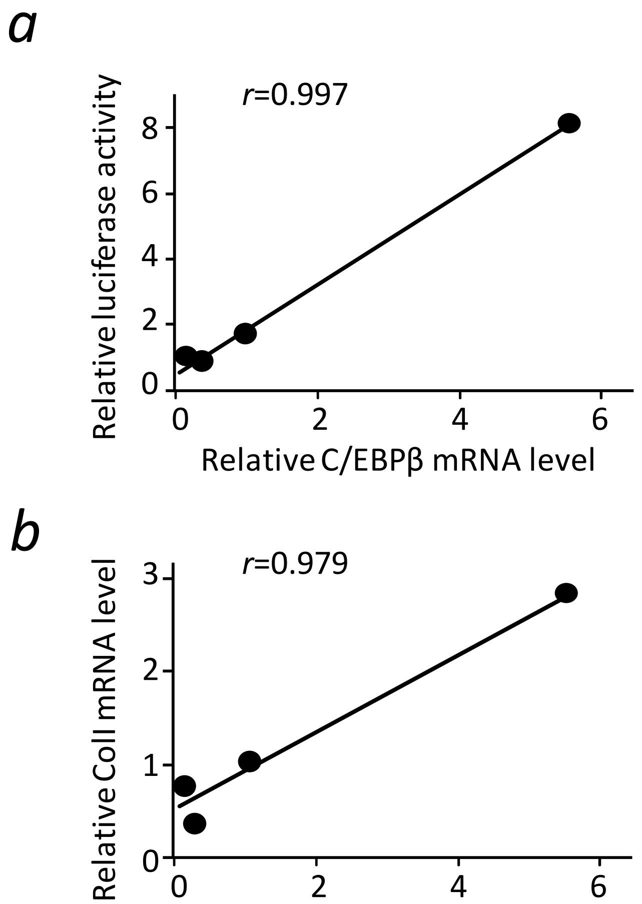

Supplementary figure S8. Saa3 promoter-luc mice were given the adenine diet for 1 week. Kidney tissues were isolated and subjected to the *ex vivo* luciferase activity assay and mRNA expression analysis (n = 4). The relative mRNA expression levels of C/EBPβ and ColI in kidney tissue were determined by quantitative PCR and normalized to L19 mRNA level (n = 4). Pearson's correlation coefficient showed a positive correlation between C/EBPβ mRNA expression level and luciferase activity (*a*) and ColI mRNA expression level (*b*) in kidney tissue.

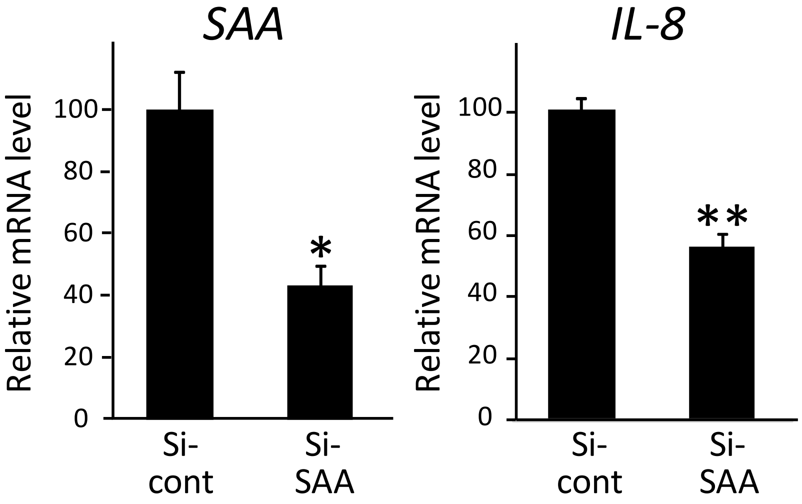

Supplementary figure S9. Effect of SAA siRNA on IL-8 mRNA expression in HK-2 cells. siRNA duplex oligoribonucleotides against human SAA (NM_000331) was synthesized by Sigma. The sequences were as follows: sense 5′-GAGAUUCUUUGGCCAUGGU-3′, antisense 5′-ACCAUGGCCAAAGAAUCUC-3′. HK-2 cells were transfected with control siRNA (Si-cont) or SAA siRNA (Si-SAA) to a final concentration of 20 nM using LipofectAMINE RNAimax (ThermoFisher Scientific). After 2 days of transfection, total RNAs were extracted and subjected to quantitative PCR analyses. The relative mRNA expression levels of SAA and IL-8 were determined by quantitative PCR and normalized to GAPDH mRNA level (n = 4). All values are expressed as mean ± S.E. Student’s t-test. *$p < 0.05$, **$p < 0.01$ compared with those of cells transfected with control siRNA (Si-Cont).

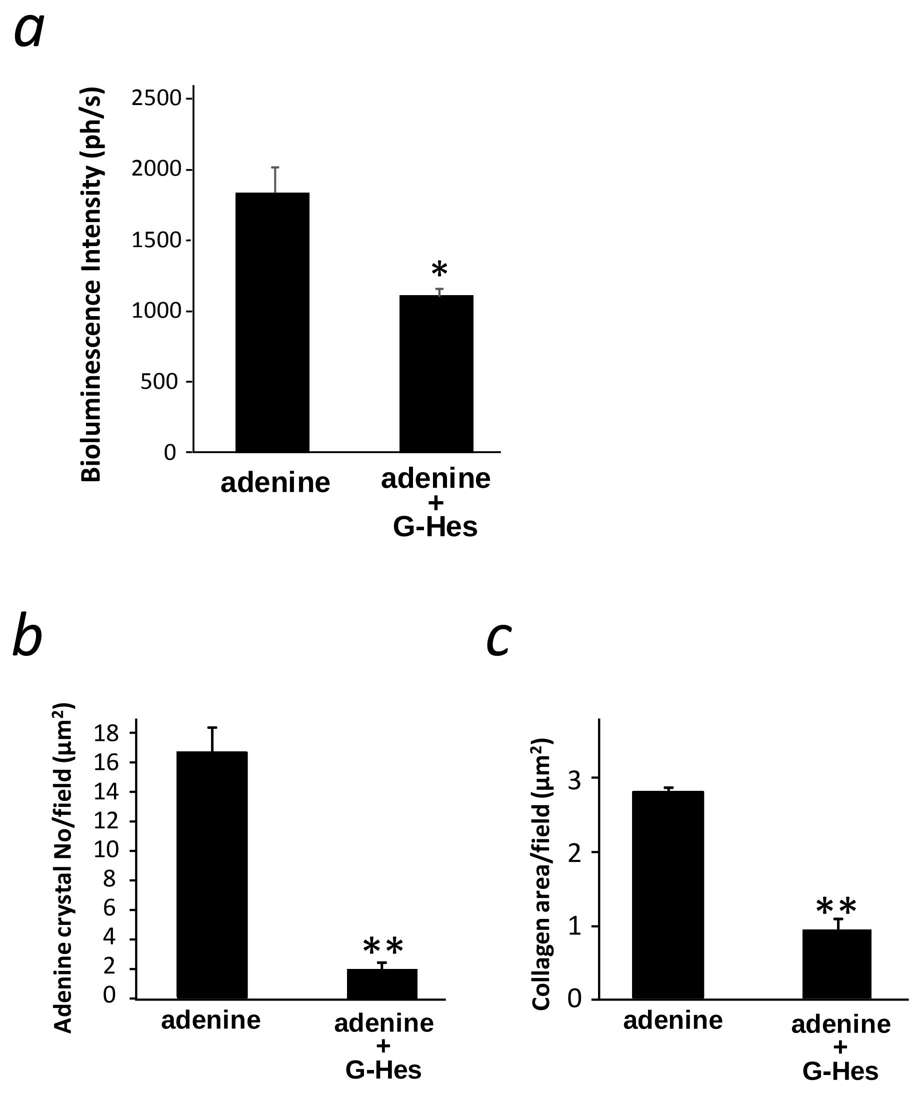

Supplementary Figure S10. Effect of glucosyl-hesperidin (G-Hes) on adenine-induced kidney disease development. Mice were divided into two groups (n = 4) receiving the adenine diet (adenine) or the G-Hes-mixed adenine diet (adenine+G-Hes). *a*, For the *in vivo* bioluminescence imaging, G-Hes was given to Saa3 promoter-luc mice receiving the

adenine diet for 1 week. Saa3-luc mice were imaged NightOWL II Imaging Systems LB983 (Berthold Technologies, Bad Wildbad, Germany) and photons emitted from tissues were analyzed using Indigo in vivo image software (Berthold). Signal intensity was quantified as the sum of all detected photon counts per second. *b*, For histopathological image analyses of the adenine-induced kidney, G-Hes was given to wild-type mice receiving the adenine diet for 3 weeks. Kidney tissues were isolated, and paraffin-embedded sections were subjected to hematoxylin and eosin (H&E) or Azan-Mallory staining. Deposition of DHA crystals in renal tubules and accumulation of collagen were calculated with randomly chosen forty fields in three slices. All values are expressed as mean ± S.E. Student's *t*-test. $*p < 0.05$, $**p < 0.01$

Supplementary figure S11.

Figure 3A

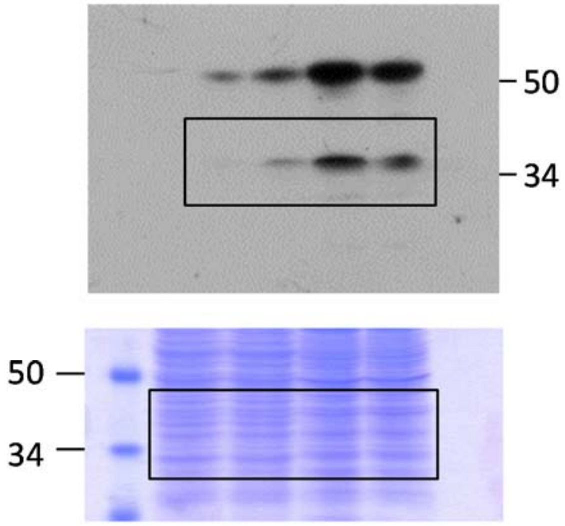

Figure 3B

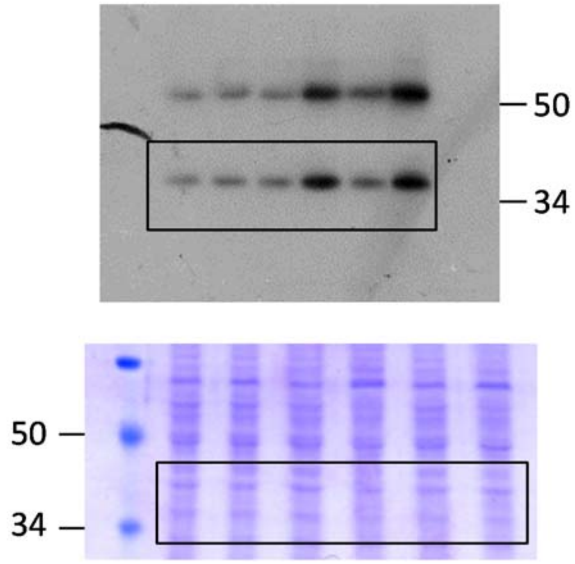

Supplement: Supplementary file 1 — Supplementary Figures [file 41598_2019_50685_MOESM1_ESM.pdf]
